# The evolution and functional repertoire of translation proteins following the origin of life

**DOI:** 10.1186/1745-6150-5-15

**Published:** 2010-04-08

**Authors:** Aaron D Goldman, Ram Samudrala, John A Baross

**Affiliations:** 1 Department of Microbiology, University of Washington, Box 357242, Seattle, WA, 98195, USA; 2 Center for Astrobiology and Early Evolution, University of Washington, Box 352192, Seattle, WA, 98195, USA; 3 School of Oceanography, University of Washington, Box 357940, Seattle, WA, 98195, USA

## Abstract

**Background:**

The RNA world hypothesis posits that the earliest genetic system consisted of informational RNA molecules that directed the synthesis of modestly functional RNA molecules. Further evidence suggests that it was within this RNA-based genetic system that life developed the ability to synthesize proteins by translating genetic code. Here we investigate the early development of the translation system through an evolutionary survey of protein architectures associated with modern translation.

**Results:**

Our analysis reveals a structural expansion of translation proteins immediately following the RNA world and well before the establishment of the DNA genome. Subsequent functional annotation shows that representatives of the ten most ancestral protein architectures are responsible for all of the core protein functions found in modern translation.

**Conclusions:**

We propose that this early robust translation system evolved by virtue of a positive feedback cycle in which the system was able to create increasingly complex proteins to further enhance its own function.

**Reviewers:**

This article was reviewed by Janet Siefert, George Fox, and Antonio Lazcano (nominated by Laura Landweber)

## Background

Proteins are the primary functional biomolecules of life. Protein synthesis is directed by translating the genetic code from informational RNA molecules. The RNA world hypothesis proposes that a simple RNA-only genetic system preceded the modern one. In the RNA world model, RNA genes direct the synthesis of functional RNA molecules rather than proteins [1]. This system may have arisen from robust protometabolic networks [2] and probably remained dependent on inorganic catalysts [3,4] and short prebiotic peptides [5] to complement the limited functional capacity of RNA. Early analyses of bacterial and archaeal genomes showed that genes and gene clusters associated with transcription and translation are indeed highly conserved while DNA replication is not [6,7].

The onset of protein translation allowed RNA genes to exert a greater degree of biochemical control by encoding the synthesis of functional proteins. The modern translation system reflects this history as one of the few metabolic processes dominated by RNA [8-10]. An amino acid sequence is encoded on messenger RNA (mRNA) and translated to protein by transfer RNA (tRNA). The ensuing peptide elongation is catalyzed by functional RNAs in the ribosome (rRNA).

**Figure 1:**
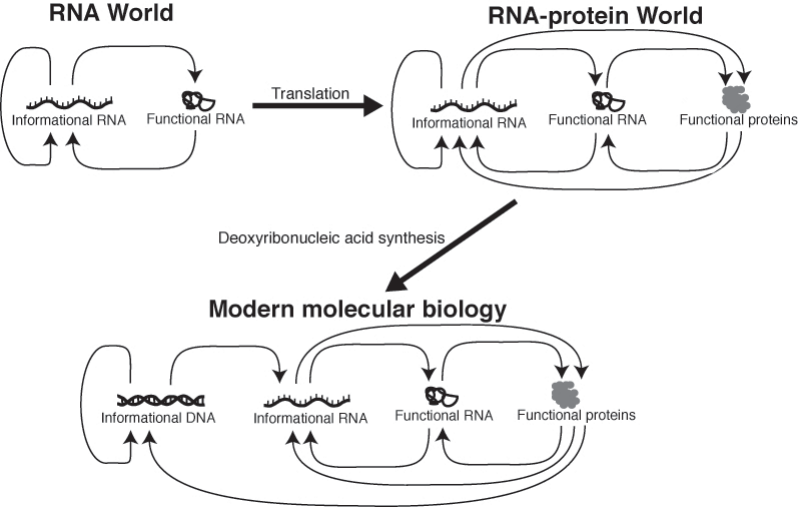
**A popular model for the development of the genetic system**. The RNA world hypothesis proposes that the first genetic system involved informational RNA molecules that encoded the synthesis of modestly functional RNA molecules [1]. Protein translation developed during this period leading to the RNA-protein world. Finally, protein enzymes produced deoxyribonucleotides through ribonucleotide reduction. The availability of deoxyribonucleotides led to the establishment of the DNA genome and the modern genetic system [5].

Several lines of evidence suggest that the onset of protein translation predated the establishment of the DNA genome [5,11]. The synthesis of deoxyribonucleotides, for example, was probably not achievable under prebiotic conditions and thus required enzymatically catalyzed ribonucleotide reduction [5,12]. In contrast, the prebiotic syntheses of both ribonucleotides [13] and amino acids [14] can occur without catalysis from biological enzymes. We note that many progressions for the origin of the genetic system have been proposed [15], however the strongest evidence supports the model described above (and illustrated in Figure 1).

The modern translation system relies on proteins to carry out several key functions. Ribosomal proteins play an important role in supporting ribosome structure and promoting translation. GTP-hydrolyzing regulatory factors help direct the initiation, elongation, and completion of translation [16,17]. Proteins are also required to charge tRNA molecules with the appropriate amino acid [18] and adjust their binding affinity to the ribosome [19]. Here we examine the early evolution of these proteins by a survey of conserved structural architectures.

Modern proteins are composed of one or more architectural folds that can function and evolve independently [20]. Protein and RNA fold modules are highly conserved in evolution [21,22]. In contrast, the evolutionary convergence of two unrelated lineages toward a common fold is thought to be rare [23]. Convergent evolution attributable to functional similarities has been observed at the level of local structural motifs, but in these cases the original overall fold architecture is maintained [24]. Thus, nonhomologous proteins that share a common structural topology will most likely represent an ancient evolutionary relationship that is too distant to be detected by sequence similarity [25]. Recent work by Wang et al. [26] established a phylogeny of protein fold architectures based on the distribution of these folds across all completed genomes. We apply ancestry values derived from this phylogeny to the experimentally determined fold architectures present in translation proteins.

## Results and discussion

### Structural evolution of translation proteins

We first observed and compared the structural evolution of three functional categories of translation proteins: translation regulatory proteins, ribosomal proteins, and tRNA-related proteins. For a given functional category, protein folds and their respective phylogenetic ancestries were identified through a combination of data from the Gene Ontology database (GO) [27], the ASTRAL database [28], and the Molecular Ancestry Network database (MANET) [29]. These data are available as additional online material (Additional file 1).

**Figure 2:**
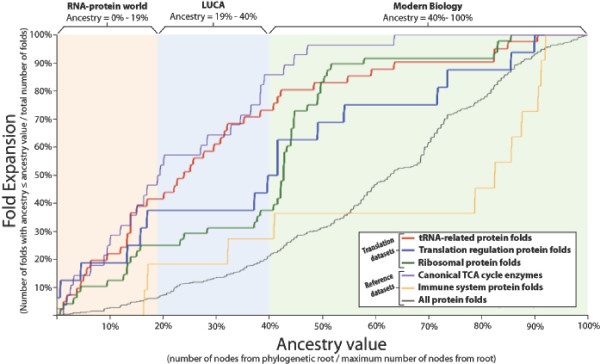
**Protein fold expansion plotted as a function of ancestry**. Fold expansion is calculated as the cumulative fraction of folds less than or equal to a given ancestry value. Ancestry values for fold architectures were derived from the phylogenetic tree of all folds by Wang et al. [26] and are equal to the number of nodes from a given fold to the root of the phylogenetic tree divided by the number of nodes from the most recent fold to the root of the tree. Fold expansion can be considered a proxy for sophistication while ancestry value can be considered a proxy for evolutionary time. For reference, the same analysis is performed on canonical TCA cycle enzymes, immune system proteins, and the whole proteome (see Results and discussion). The first fold of a ribonucleotide reductase catalytic domain appears at 19% ancestry, while the first fold found in only one taxonomic domain of life appears at 40% ancestry. We use these values to approximate ranges in ancestry value that correspond to the RNA-protein world, the era of the Last Universal Common Ancestor (LUCA), and the era of modern biology. These results reveal a rapid expansion of translation protein architectures before the divergence of LUCA and even before the establishment of the DNA genome. Quantitative features of these results are presented in Table 1.

We observed the structural evolution of each category of translation proteins by calculating its fold expansion as a function of ancestry value. Nonredundant sets of all folds found in all proteins were created for each category. The phylogenetic ancestry value of each fold was calculated by Wang et al. [26] as the number of nodes from that fold to the root node divided by the number of nodes from the most recent fold to the root node. The ancestry value can be considered a proxy for relative age where 0% is the most ancient value and 100% is the most recent value. Fold expansion is calculated for a given functional category as the number of folds equal to or less than a given ancestry value divided by the total number of folds. Fold expansion can be considered a proxy for functional sophistication, where 100% represents the current level of sophistication. Figure 2 shows fold expansion plotted as a function of ancestry and thus illustrates the increase in sophistication over time for the three categories of translation proteins.

Three additional datasets are analyzed for comparison. Recently, a superimposition of metabolic pathways was used to identify a canonical TCA cycle [30] that is proposed to reflect the core metabolism of the Last Universal Common Ancestor to all extant life (LUCA). The enzymes that catalyze the supposedly ancient reactions within this canonical TCA cycle were used to illustrate an extremely accelerated fold evolution. In contrast, the full set of immune system proteins was used to demonstrate a slower evolutionary expansion given that this category is unlikely to have any relevance to the origin of life. The overall expansion of the proteome is also shown.

All three categories of translation proteins show a significantly earlier structural expansion than the expansion of the immune system proteins or the whole proteome (Figure 2). tRNA-related proteins show the earliest structural expansion followed by translation regulatory factors, then ribosomal proteins. The earliest fold catalyzing ribonucleotide reduction is found at an ancestry value of 19%. This ancestry value is used to mark the transition from an RNA-protein system to a DNA-RNA-protein system. Wang et al. [26] determined that the first folds found only in a single taxonomic domain appear at 40% ancestry. This ancestry value is used to identify the divergence of LUCA into the three domains of life. Thus we are able to classify three periods of proteome development: the RNA-protein world (0%-19% ancestry), the era of LUCA (19%-40% ancestry), and the era of modern biology (40%-100% ancestry). Quantitative features of fold expansion within these three periods are summarized in Table 1. This analysis reveals an early development of translation proteins and a particularly rapid development of tRNA-related proteins during the RNA-protein world and the era of LUCA.

**Table 1:** Quantitative features of fold expansion curves presented in Figure 2.

Statistic	Protein category			ancestry ≤ 19% (prior to DNA genome)		ancestry ≤ 40% (prior to divergence of LUCA)		ancestry ≤ 100% (all protein folds)
	tRNA-related proteins			41.5%		72.2%		100%
	
	Regulation of translation			37.5%		50.0%		100%
	
**Final fold**	Ribosomal proteins			25.0%		37.5%		100%
**expansion**								
	Canonical TCA enzymes			50.0%		85.7%		100%
	
	Immune system proteins			18.2%		27.3%		100%
	
	Whole proteome			6.5%		21.0%		100%

	tRNA-related proteins			4.2%		16.7%		70.6%
	
	Regulation of translation			3.9%		12.0%		60.3%
	
**Area**	Ribosomal proteins			2.5%		8.8%		62.1%
**under**								
**curve**	Canonical TCA enzymes			4.7%		18.0%		77.0%
	
	Immune system proteins			0.4%		5.0%		35.1%
	
	Whole proteome			0.5%		3.3%		40.3%

### Functional capacity of the primitive translation system

**Figure 3:**
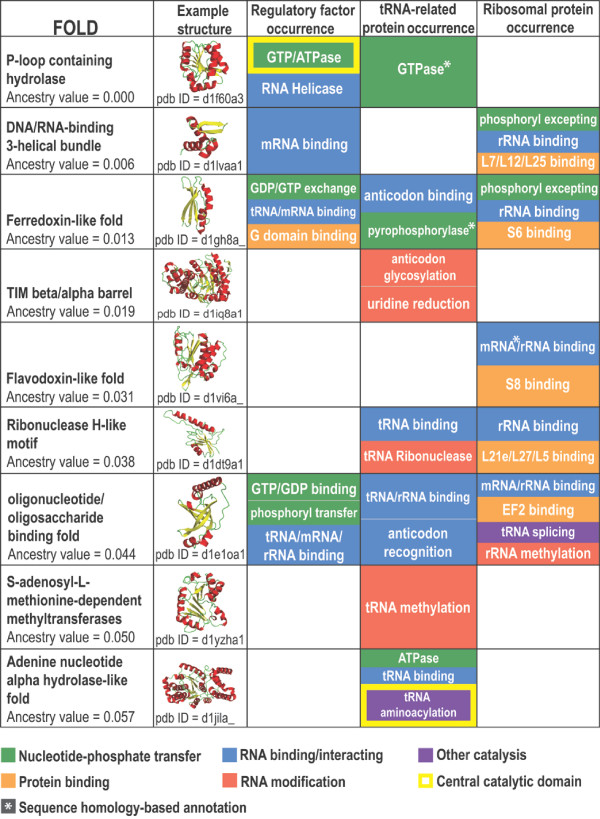
**A summary of functional annotation of the most ancestral translation protein folds**. Nine of the ten most ancestral folds identified by Wang et al. [26] are present in translation proteins. The specific functional roles of these folds converge on four general categories: high energy phosphoryl transfer, RNA modification, RNA binding, and protein binding. Exceptions are aminoacylation by tRNA synthetase and tRNA splicing by ribosomal protein S28e. Taken together, the functions imparted by these nine most ancestral folds represent all of the central protein functions in the modern translation system (Figure 4). A summary of the genes in which these folds are found is available as Additional file 2. A detailed annotation of functions imparted by these folds is available as Additional file 3.

**Figure 4:**
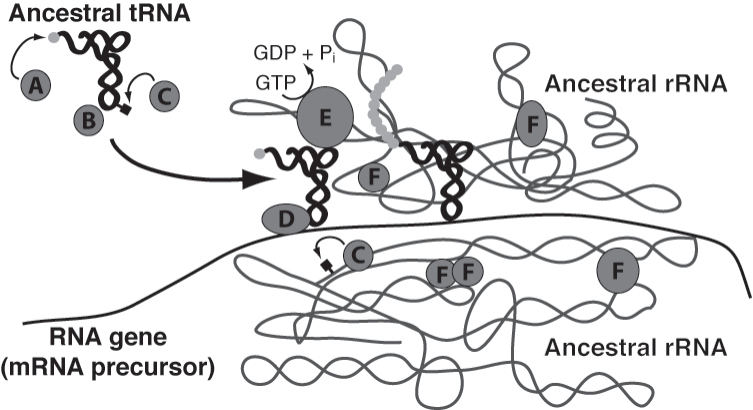
**A model of protein enhancement in the primitive translation system**. The protein functions illustrated here are imparted by the earliest translation protein fold architectures and are summarized in Figure 3. A) The ancestor of the class I aminoacyl tRNA synthetase (ARS) catalytic domain charges tRNAs with amino acids (lowest ancestry value = 5.7%). B) An ancestor of a noncatalytic ARS domain binds tRNA anticodon and interacts with protein "A" during aminoacylation of the tRNA (lowest ancestry value = 1.3%). C) Ancestors of RNA modification enzymes add small organic molecules to tRNA and rRNA to adjust mutual binding affinity (lowest ancestry value = 1.9%). D) Ancestors of regulatory factor domains bind mRNA and tRNA to stabilize their interaction during peptide chain initiation and elongation (lowest ancestry value = 1.3%). E) Ancestors of the regulatory factor GTPases drive peptide elongation forward and sensitize the ribosome to codon-anticodon mismatches (lowest ancestry value = 0.0%). F) Ancestors of ribosomal proteins are able to bind rRNA and one another to stabilize the primitive ribosome complex (lowest ancestry value = 0.6%). These functions were all present before 6% ancestry, indicating that a robust translation system existed early on in the RNA-protein world.

Nine out of the ten most ancestral fold architectures were found in translation proteins. The molecular functions imparted by these folds were annotated through a combination of data from the NCBI Conserved Domains Database (CDD) [31] and literature review. A summary of these functions is presented in Figure 3. A summary of the genes in which these folds are found and a fully annotated list of these functions are available as additional online material (Additional files 2 and 3, respectively). Nearly all of these folds converge on four basic functions: nucleotide-phosphate transfer, RNA binding, protein binding, and RNA modification. Amongst these folds are two noteworthy catalytic domains. The most ancestral fold (P-loop containing hydrolase) is ubiquitous in regulatory proteins as a GTPase domain [32]. The tenth most ancestral fold (adenine nucleotide alpha hydrolase-like fold) is found as the conserved catalytic domain of all class I tRNA synthetases [33]. These ancestral folds were likely present as single domain proteins early on in the RNA-protein world. A model of translation protein functions during the RNA-protein world was developed using these annotations (Figure 4).

The majority of these ancestral fold functions promote the modern translation system through binding to other components of the translation apparatus. Five ancestral folds are present as single domain ribosomal proteins with the ability to bind RNA and other proteins (DNA/RNA binding 3-helical bundle, Ferrodoxin-like fold, Flavidoxin-like fold, Ribonuclease H-like motif, and Oligonucleotide/oligosaccharide binding fold). Three ancestral folds found in regulatory factors also have the ability to bind RNA (DNA/RNA binding 3-helical bundle, Ferrodoxin-like fold, and Oligonucleotide/oligosaccharide binding fold). In one case, the fold simultaneously binds tRNA and mRNA (Ferrodoxin-like fold). In another case, the fold simultaneously binds tRNA, mRNA, and rRNA (Oligonucleotide/oligosaccharide binding fold). These folds likely played an important role in stabilizing the ribosome and coordinating the mRNA:tRNA:rRNA complex in the primitive translation system.

Many ancestral fold functions also contribute to the fidelity of the modern translation system. In the modern translation apparatus, codon-anticodon mismatches cause the ribosome to take a suboptimal structural conformation and the elongation factor GTPase to hydrolyze additional GTPs [19]. These alterations generally force a mismatched tRNA to dissociate from the translation apparatus without adding its amino acid to the peptide chain. tRNAs are regularly modified by the addition of small organic molecules in order to give each tRNA the same binding affinity to the rRNA, thus assuring the same proofreading potential for each amino acid [19]. The rRNA binding, GTPase, and RNA modifying functions imparted by these ancestral folds may have played an important role in allowing the primitive translation apparatus to prevent incorrect codon-anticodon binding.

It is possible that these results may be confounded by the recent exaptation of translation protein functions from an unrelated molecular network [34]. Given, however, that translation is a highly conserved and ancient process [35], it is more likely that protein functions would originate within the translation network and be exapted to another more recent network rather than the other way around. Furthermore, these most ancient proteins would probably have had a generalized function. Specification to a single network node would have come later in the development of the proteome. In addition, the majority of these folds are represented by a number of domains with disparate functions (see Additional file 3) and thus are reasonably robust such that we can discount exaptation as having only a minor effect on our analysis.

## Conclusion

**Figure 5:**
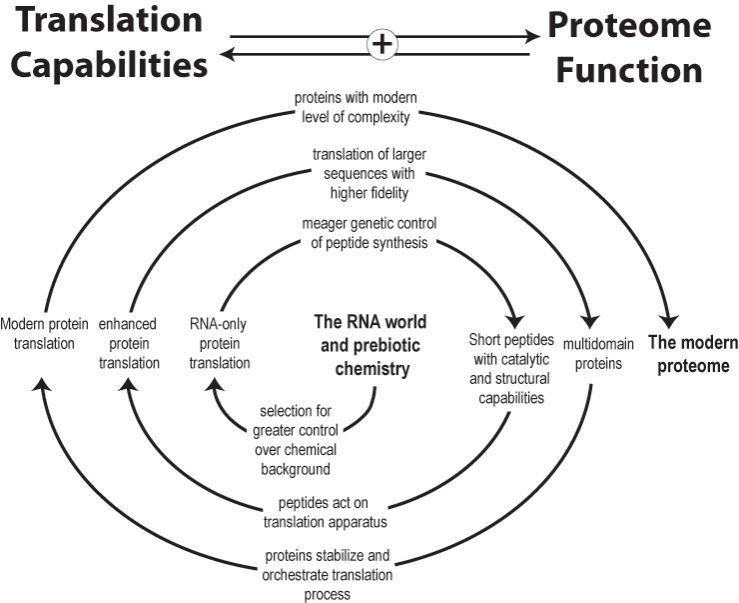
**A positive feedback loop mechanism for the early development of the translation system**. At each evolutionary stage, the stability and fidelity of the translation system is enhanced by the peptides it produces. This new superior translation system is able to synthesize proteins of even greater functional capability that can, in turn, act on the translation system to further enhance its own functional capability. This mechanism may have been a central driving force in the transition from the RNA world to modern cellular life.

This survey of translation protein folds demonstrates that all of the major functions required for a stable and capable translation system were present very early on during the development of the RNA-protein world. Our analyses suggest that translation proteins underwent major evolutionary expansion well before the first species diverged from LUCA and even before the DNA genome was established. The original RNA-only translation system undoubtedly became increasingly efficient and accurate due to enhancement by the peptides it produced. This enhanced translation system would allow for the synthesis of more complex proteins. These superior proteins could once again act on the translation system to further improve its own functional capabilities. The initial onset of translation could thus have produced a positive feedback cycle that accelerated its own evolution (Figure 5). The transition from a primitive translation system to a sophisticated one may have been not only rapid but also deterministic.

## Methods

### Acquiring datasets

Fold architectures from translation proteins and reference category proteins were identified using the Gene Ontology database (GO) [27,36] in combination with hand annotation. GO results were filtered for proteins with known structures entered in the Protein Data Bank (PDB) [37,38]. The PDB IDs for each protein were cross-referenced with the 40% redundant ASTRAL database [28,39,40] in order to identify the folds within each protein. The fold data were then cross-referenced with fold ancestry values from the Molecular Ancestry Network (MANET) database [29,41]. Separate datasets were created for three functional categories of translation proteins and three reference categories (see Results and discussion). These datasets are available as Additional file 1. For each functional category, a nonredundant set of all folds found in all proteins was created for fold expansion analysis.

### Analysis

Ancestry values were derived by Wang et al. [26] using their phylogenetic tree of all protein folds. The ancestry value is equal to the number of nodes from a given fold to the root of the tree divided by the number of nodes from the most recent fold to the root of the tree. For each functional category, fold expansion was calculated as the cumulative fraction of folds with respect to ancestry value. That is, at a given ancestry value, the fold expansion is equal to the number of folds with an ancestry value less than or equal to the given ancestry value divided by the total number of folds in the functional category. Functional annotation of folds was performed by a combination of NCBI Conserved Domains Database (CDD) [31,42] searches and literature review.

## List of abbreviations

ARS: Aminoacyl tRNA Synthetase; CDD: Conserved Domains Database; GO: Gene Ontology; LUCA: Last Universal Common Ancestor; MANET: Molecular Ancestry NETworks database; PDB: Protein Data Bank.

## Competing interests

The authors declare that they have no competing interests.

## Authors' contributions

ADG designed and implemented this study and wrote this manuscript. RS and JAB assisted in the design of this study and the preparation of the manuscript. All authors read and approved the manuscript.

## Reviewer's report 1

Dr. Janet Siefert, Rice University, Department of Statistics, Houston, TX USA

### Reviewer's Comments

1. Is the question posed new and well defined?

The question is very well defined. The question itself is not new, just unsolved.

2. Are the methods appropriate and well described, and are sufficient details provided to replicate the work?

The methods used by the authors are what makes the approach to an old question relevant for investigation. After the methods of Wang et al, [26] they utilize a composite architectural protein motif phylogenetic approach to place translational proteins and appearance of DNA within the origin of life timeline provided by the Wang etal efforts.

3. Are the data sound and well controlled?

The data is typical of extant genome content, annotation, and analyses. The critical assumption in this work is that parsimony is an accurate model for tracking the evolution of protein fold expansion and reduction in current sequenced genome architectures. As mentioned, this is a specific application of the model and analyses performed by Wang et al. [26]. Under these analyses conditions, this seems to be a reasonable model. The level of resolution - architecture and structure and presence or absence of fold - is likely to be observed when major changes occur as opposed to minor changes (eg as in sequence), and should therefore likely to reflect a more parsimonious path when using genomes for retrospection. Using the protein fold data to determine the ancient history of translation proteins represents sound data and date under control.

4. Does the manuscript adhere to the relevant standards for reporting and data deposition?

The manuscript adheres to reporting and data deposition standards.

5. Are the discussion and conclusions well balanced and adequately supported by the data?

The authors conclude that translation proteins are early developing and that there was a particularly rapid development of tRNA protein prior to the invention and use of DNA. They support their case through the results from their analyses, the predominance of described "ancient protein folds" in these translation proteins, and the identification of these folds in proteins inherent to ribosomal functions including high energy transfer, RNA and protein binding and RNA modification. They provide an alternate hypothesis to their conclusion involving speculation that some unknown molecular network might have produced their results, but conclude that this is unlikely given the universal and ancient nature of the translation system this is unlikely.

6. Do the title and abstract accurately convey what has been found?

The title and abstract are appropriate.

7. Is the writing acceptable?

The writing is straightforward and engaging to read. It doesn't hurt that the subject and question while old, are engaging in themselves.

I declare that I have no competing interests.

## Reviewer's report 2

Dr. George Fox, Department of Biology and Biochemistry, University of Houston, Houston, TX USA

### Reviewer's Comments

The contribution by Goldman et al. expands our understanding of the importance of the early development of translation during the final stages of transition to the modern post-LUCA world and is significant and pending significant revision is worthy of publication. The authors examine ribosomal components from the perspective of fold ancestry using an approach that has previously been pioneered by Gustavo Caetano-Anolles, Jay Mittenthal and their colleagues [26]. In particular, the extent to which the ten folds with the lowest ancestry scores are associated with ribosomal components and functions is examined. Of special interest is the reasonable speculation presented in which specific ancestry values are associated with the key transitions between the early RNA World, LUCA, and modern biology. If these are reasonable values then one obtains significant insight to what the status of things were at the time of these key transitions in evolutionary history. Although the paper is an important contribution, it will require significant revision in several areas. The most significant omission is supporting documentation. In particular, readers should be provided with a list of which ribosomal components were considered in each of the three categories, the folds they contain and the ancestry value of those folds. This table can be provided as supplementary material. The existing supplementary material actually provides this information in a very indirect manner but the proposed table would greatly simplify matters.

### Author's Response

We have followed this request and have now made such a table available as Additional file 1.

### Reviewer's Comments

The paper creates, perhaps unwittingly, the impression that ancestry values equate strongly with actual historical age. The ancestry values are based on the phylogenetic distribution of the fold and the extant to which the fold is put into use in a variety of places, *e.g*. its expansion. However, the first peptides made by the primitive translation system were likely relatively simple and hence perhaps not amenable to "expansion" because they actually had limited utility. Thus, for example, ribosomal proteins such as L3 and L4 have folds that are seldom if ever used elsewhere but nevertheless these proteins and hence their folds are likely to be very ancient (see [43]). Because these folds are universal among ribosomal proteins in all three Domains of life, they will still get rather low ancestry scores but will not necessarily be among the lowest where they might actually belong from the historical perspective. Thus, the reader should be cautioned about this potential limitation of the ancestry values.

### Author's Response

Actually, in the fold phylogeny created by the Caetano-Annoles group that is used in this study, fold ancestry is determined by the number of genomes in which a fold is found rather than the number of times a fold appears in a genome. Thus, such folds as described by Dr. Fox would still be identified as ancient if they appeared in every genome, even if only once per genome.

### Reviewer's Comments

The figure entitled "A summary of functional annotation of the most ancestral translation protein folds" is also mildly problematic in that it sometimes tabulates function and sometimes specific components. Thus, for example under ribosomal protein occurrence we find "peptidyl transfer". This apparently refers to one of the r-proteins near the PTC site (L2!?) but in fact none of the proteins are thought to be involved with peptidyl transferase activity at all, which instead is widely held to be associated with the large subunit rRNA.

### Author's Response

This was a poor annotation that has been eliminated. The original annotation was referring the observation that the His229 of L2 promotes (but does not catalyze) peptidyl transfer [44].

### Reviewer's Comments

In other places, specific proteins are mentioned, e.g. "S6 binding". It would greatly simplify things if an additional table were provided that indicated which ribosomal genes were associated with each of these 9 low ancestry folds of this figure. This additional table might partially overlap in content with the other additional table requested above. Perhaps the table suggested here would be in main manuscript and the other larger table in supplementary materials.

### Author's Response

We have created this table and made it available as Additional file 2. This table is actually larger than Figure 3 because each fold is encoded within many different translation genes. It should be noted that most of these genes encode several different folds. So, for example, a fold found in an enzyme does not necessarily impart the catalytic function of the enzyme.

### Reviewer's Comments

Other minor problems include (1) The manner in which the figure entitled "protein fold expansion plotted as function of ancestry" was obtained is rather confusing. A stronger explanation is needed as to what data was used to construct this figure and how it was utilized;

### Author's Response

We followed this request and have bolstered our explanation of this analysis in both the Results and discussion and Methods sections.

### Reviewer's Comments

(2) The figure entitled "A model for the development of the modern genetic system from the RNA world" is a simple recapitulation of the usual, though not necessarily completely correct, view of a progression from the RNA World, to a RNA protein-World to ultimately modern organisms. This is not conceptually difficult, nor original, and the figure contributes nothing new and therefore is not needed;

### Author's Response

We absolutely did not intend to take credit for this model and cite several sources in which it was previously proposed. We opt, however, to keep this figure in the main article as we feel its value as an illustrative tool broadens the readership of the manuscript. We have altered the introduction to both eliminate any confusion over the credit for this model and acknowledge that other models for the origin of the genetic system have been proposed.

### Reviewer's Comments

(3) The discussion is unnecessarily vague in multiple places. For example, in the second paragraph of the section entitled "functional capacity of the primitive translation system" we see the statement "Three ancestral folds found in regulatory factors have the ability to bind RNA." What are these 3 folds? There are several places in the discussion where such useful additional information can be readily provided;

### Author's Response

We have amended this discussion and now list the specific folds to which we refer when making these statements.

## Reviewer's report 3

Dr. Antonio Lazcano, Departamento de Biología, Universidad Nacional Autónoma de México, Ciudad de México, Distrito Federal, Mexico

### Reviewer's Comments

This is a good, rather interesting paper that approaches the issue of the relative antiquity of different component of modern cells in a very original fashion. What the authors have made a valuable contribution by estimating the relative antiquity of basic cellular processes in the absence of other types of evidence that could allow a precise dating. This approach is somewhat similar to the estimates of relative ages determined by paleontologists and is deeply rooted in evolutionary biology. I endorse its publication, but would like to offer a few suggestions that the authors and editors may want to consider.

This work has been written in a rather sober style that may lead to some misunderstandings. Many would object, for instance, to the claim that "Proteins are the primary functional biomolecules of life", since the same would apply to lipids, for instance.

### Author's Response

Lipids have important functional roles in the compartmentalization of cellular components, the storage of chemical energy, and intercellular communication. However, we do not agree that they stand on equal footing with proteins as the "primary functional biomolecule of life". Unlike lipids, proteins exhibit a broad range of functional activities and, via translation, are the direct result of gene expression. In addition, the functional activities of lipids described above are all produced, maintained, and regulated by proteins.

### Reviewer's Comments

The title, in fact, may reflect a slight confusion on what is meant by the origin of life and the distinction between this event and later processes that may be the outcome of Darwinian evolution acting over protein-free, RNA-dependent living systems.

### Author's Response

While we prefer to view the origin of life as a process that began with the formation of the solar system and ended with the divergence of the Last Universal Common Ancestor, the common view is that the origin of life was a single event in time. We have altered the title to alleviate any confusion caused by our alternate perspective.

### Reviewer's Comments

Goldman et al make a rather splendid one-paragraph summary of what may have been an RNA world dependent on inorganic catalysts and prebiotic polypeptides, and that protein synthesis first evolved in an RNA world. Indeed, as underlined by Kumar and Yarus [45], four of the central reactions involved in protein biosynthesis are catalyzed by ribozymes, and their complementary nature suggests suggestive that they may have first appeared in the RNA world. However, this is independent from the proposal that translation predated the emergence of DNA genomes. Moreover, while it is true that the elegant synthesis of pyrimidine ribonucleotides reported by Powner et al. [13] has led to new understanding in the chemistry of nucleobases under possible primitive conditions, the appearance and accumulation of the polyribonucleotide molecules required for the RNA world from a prebiotic soup remains an open question. This issue is, of course, further complicated by the chemical lability of RNA molecules. The nature of the predecessor(s) of the RNA world (if such predecessors actually existed) are completely unknown and can only be surmised.

### Author's Response

Dr. Lazcano makes a good point that the prebiotic synthesis of RNA molecules is not requisite for the validity of the RNA world hypothesis. What's more, it would be hubristic to maintain that RNA synthesis in the RNA world occurred exactly as demonstrated by any laboratory synthesis. That said, we only mention this work as one of several independent lines of evidence supporting the notion that RNA preceded DNA as the central genetic molecule.

### Reviewer's Comments

The paper by Goldman et al is strongly dependent on two major assumptions. One of them is what the authors have termed "ancestry value", and I find their approach valid. The other major premise is that the earliest protein fold involved in ribonucleotide reduction has an ancestry value of 19%. In fact, the evolutionary conservation of components of the translation apparatus, together with that of other molecules involved in RNA metabolism [46] supports the contention that proteins first evolved in systems in which RNA played a major role in catalysis. However, Goldman et al may want to consider that it has also been suggested that DNA genomes predate the emergence of translation, i.e., that the evolutionary sequence was actually RNA world -> a RNA+DNA world -> DNA/RNA/protein world. While I personally consider this unlikely, mention to this possibility should be given in the text.

### Author's Response

These points are well taken. An extensive review of all proposed sequences for the origin of the genetic system is beyond the scope of this article. We suggest Dworkin et al. [15].

### Reviewer's Comments

In contrast with other energetically favorable biochemical reactions (such as hydrolysis of the phosphodiester backbone, or the transfer of amino groups), the direct removal of the oxygen from the 2'-C ribonucleotide pentose ring to form the corresponding deoxy-equivalents is a thermodynamically much less-favored reaction. This is a major constraint that strongly reduces the likelihood of multiple, independent origins of biological ribonucleotide reduction, a possibility that will raised by some. In fact, although the demonstration of the monophyletic origin of ribonucleotide reductases (RNR) is greatly complicated by their highly divergent primary sequences and the different mechanisms by which they generate the substrate 3'-radical species required for the removal of the 2'-OH group. However, sequence analysis and biochemical characterization of RNRs from the three primary biological domains has confimed their structural similarities, which speaks of their ultimate monophyletic origin. This supports the contentions and conclusions made by Goldman et al., whose paper I strongly recommend for publication.

## Reviewer comments on the final manuscript

Dr. Janet Siefert

This looks good to me and I agree to publishing as revised.

Dr. George Fox

Fine with me. Really like Additional file 2.

Dr. Antonio Lazcano

I feel perfectly happy with the changes and comments you have made. I would be very happy to see this paper published as soon as possible.

## Supplementary Material

Additional file 1 **Supplementary Data 1**. Tab-delimited tables of all folds from translation proteins with known structures organized by functional category. Click here for file

Additional file 2 **Supplementary Table 1**. A summary of all translation genes within which the ten most ancient folds are encoded. Click here for file

Additional file 3 **Supplementary Data 2**. The full list of functions imparted by ancestral fold architectures summarized in Figure 3 including complete references. Click here for file
