# Supplementary material for: The evolution and functional repertoire of translation proteins following the origin of life
Source: Biol Direct. 2010 Apr 8;5:15. doi: 10.1186/1745-6150-5-15 (PMC2873265; doi:10.1186/1745-6150-5-15)
Supplement: Additional file 2 — Supplementary Table 1. A summary of all translation genes within which the ten most ancient folds are encoded. [file 1745-6150-5-15-S2.PDF]

| FOLD | Example structure | Regulatory factor occurence | tRNA-related protein occurence | Ribosomal protein occurence |
|---|---|---|---|---|
| **P-loop containing hydrolase**<br>Ancestry value = 0.000 | 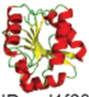<br>pdb ID = d1f60a3 | Elongation factor SelB<br>Elongation factor Tu<br>Elongation factor G<br>Elongation factor eEF-1alpha<br>Elongation factor 2<br>Initiation factor IF2/eIF5b<br>Initiation factor eIF2<br>Initiation factor 4a<br>Sulfate adenylate transferase subunit cysN/C | MnmE GTPase | |
| **DNA/RNA-binding 3-helical bundle**<br>Ancestry value = 0.006 | 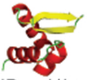<br>pdb ID = d1lvaa1 | Elongation factor SelB<br>Initiation factor 3 - eIF3k | | Ribosomal protein S18<br>Ribosomal protein L11<br>Ribosomal protein S17e |
| **Ferredoxin-like fold**<br>Ancestry value = 0.013 | 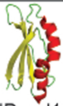<br>pdb ID = d1gh8a_ | Elongation factor aEF-1beta<br>Elongation factor eEF-1alpha<br>Elongation factor G | Phenylanine-tRNA synthetase<br>tRNA nucleotidyltransferase<br>Glutamyl tRNA-reductase | Ribosomal protein S10<br>Ribosomal protein S6 |
| **TIM beta/alpha barrel**<br>Ancestry value = 0.019 | 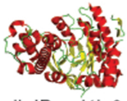<br>pdb ID = d1iq8a1 | | Queosine tRNA-guanine transglycosylase<br>Archaeosine tRNA-guanine transglycosylase<br>Putative flavin oxidoreducatase | |
| **Flavodoxin-like fold**<br>Ancestry value = 0.031 | 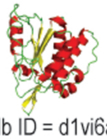<br>pdb ID = d1vi6a_ | | | Ribosomal protein S2 |
| **Ribonuclease H-like motif**<br>Ancestry value = 0.038 | 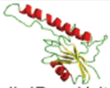<br>pdb ID = d1dt9a1 | | Ribonuclease D | Ribosomal protein L18<br>Ribosomal protein S11 |
| **oligonucleotide/ oligosaccharide binding fold**<br>Ancestry value = 0.044 | 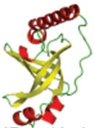<br>pdb ID = d1e1oa1 | Initiation translation factor 5a<br>eIF5a homologue (Hex1)<br>Elongation factor P<br>Initiation factor 1<br>Initiation factor 1a - aIF1a/eIFa<br>Initiation factor 2alpha | Aspartyl-tRNA synthetase<br>Phenylanine-tRNA synthetase ß<br>tRNA-binding protein TRBP111<br>Lysyl-tRNA synthetase | Ribosomal protein S12<br>Ribosomal protein S17<br>Ribosomal protein L2<br>Ribosomal protein S28e<br>rRNA (Uracil-5-)-methyltransferaseRumA |
| **S-adenosyl-L-methionine-dependent methyltransferases**<br>Ancestry value = 0.050 | 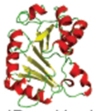<br>pdb ID = d1yzha1 | | Probable methyltransferase Rv2118c<br>Fibrillarin homologue<br>tRNA (guanine-N(7)-)-methyltransferase TrmB | |
| **Adenine nucleotide alpha hydrolase-like fold**<br>Ancestry value = 0.057 | 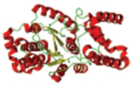<br>pdb ID = d1jila_ | | Lysyl-tRNA synthetase<br>Glutaminyl-tRNA synthetase<br>Cysteinyl-tRNA synthetase<br>Isoleucyl-tRNA synthetase<br>Arginyl-tRNA synthetase<br>Valyl-tRNA synthetase<br>Leucyl-tRNA synthetase<br>Tyrosyl-tRNA synthetase<br>tRNA-Ile-lysidine synthetase<br>TilS-like protein Aq_1887<br>Tryptophanyl-tRNA synthetase<br>Glutamyl-tRNA synthetase<br>Glutamyl-Q tRNA-Asp synthetase<br>Methionyl-tRNA synthetase | |
